# Supplementary material for: The Dual-Mode Transition of Myofibroblasts Derived from Hepatic Stellate Cells in Liver Fibrosis
Source: Int J Mol Sci. 2023 Oct 23;24(20):15460. doi: 10.3390/ijms242015460 (PMC10607848; doi:10.3390/ijms242015460)
Supplement: Supplementary file 1 [file ijms-24-15460-s001.zip › ijms-2652007-supplementary.pdf]

Supplementary Materials

This file includes:

Figure S1-3, Tables S1

Fig.S1

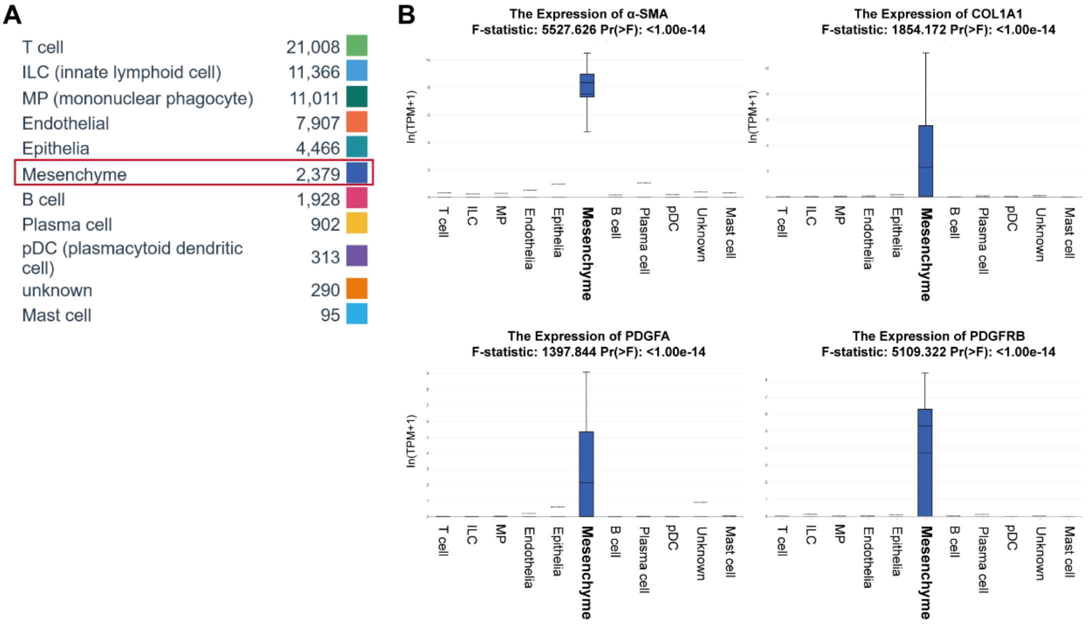

Figure S1. Cell subpopulation with myofibroblast characteristics in the single cell assay database. A. Classification of cell subpopulations in the Homo sapiens fibrosis liver single-cell database. **B**. The α-SMA, COL1A1, PDGFA, and PDGFRB expression in cell subpopulations in a single cell database.

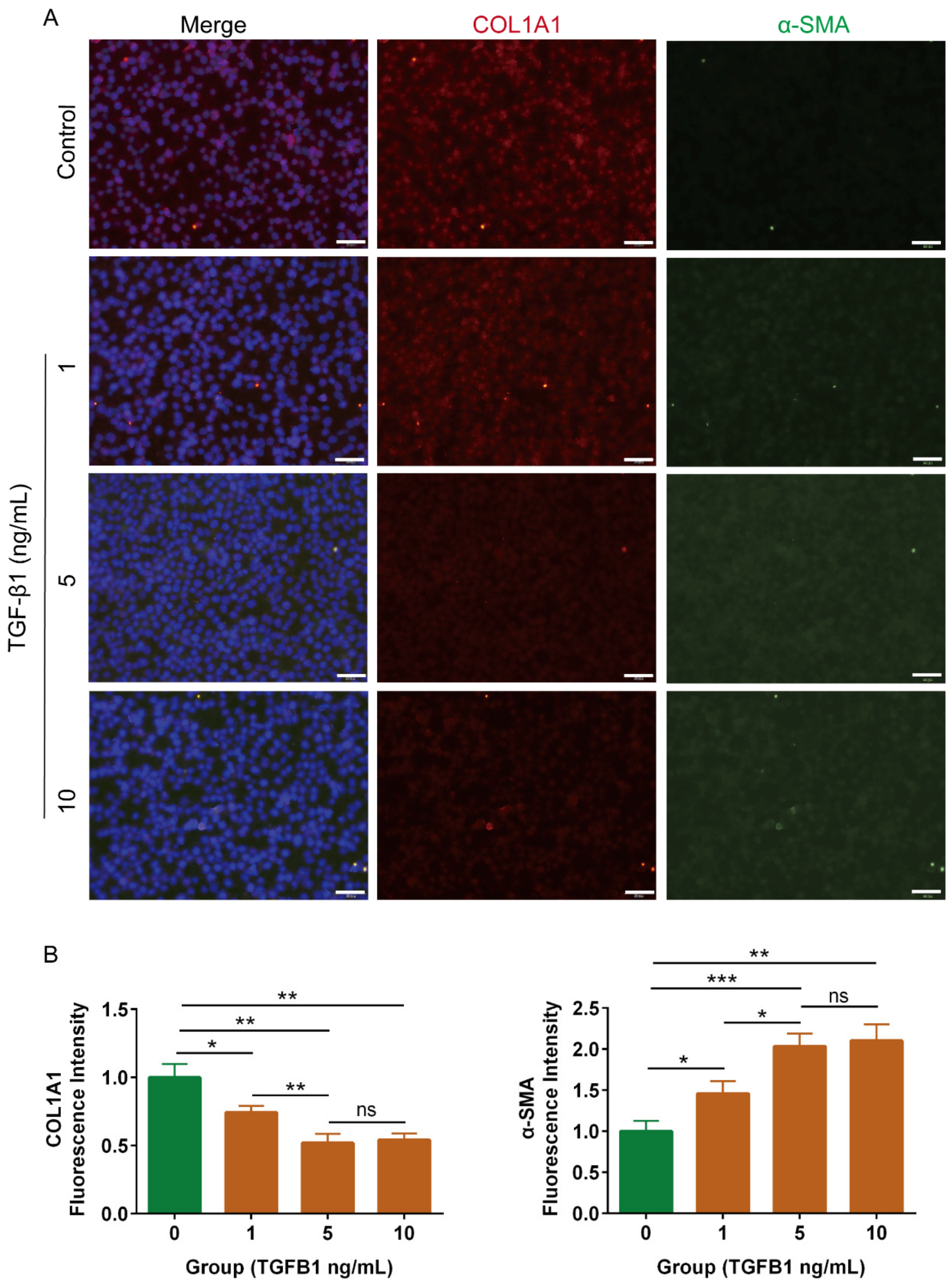

Figure S2. The protein expression of α-SMA in HSC-T6 derived myofibroblasts in the presence of gradient TGF-β1. A. The expression of α-SMA (green) and COL1A1 (red) in HSCs derived myofibroblasts was labeled with double staining; scale bar, 50 μm. B. Fluorescence intensity quantification; Image J was used for

quantification. t-tests were used for statistical analysis. *P<0.05; **P<0.01; ***P<0.001.

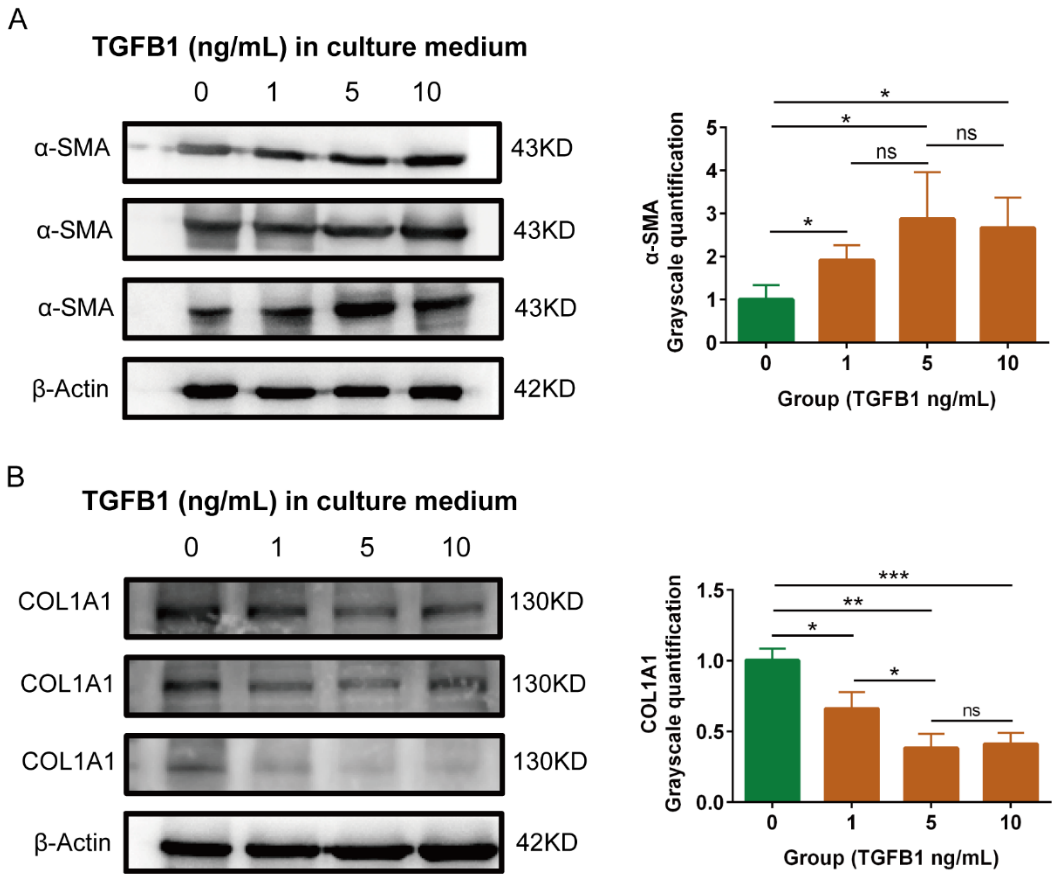

Figure S3. Cell subpopulation with myofibroblast characteristics in the single cell assay database. A. The protein expression of α-SMA in HSC-T6 derived myofibroblasts in the presence of gradient TGF-β1; The results of three independent experiments were presented; Image J was used for grayscale quantification. **B**. The protein expression of COL1A1 in HSC-T6 derived myofibroblasts in the presence of gradient TGF-β1; The results of three independent experiments were presented; Image J was used for grayscale quantification. t-tests were used for statistical analysis. *P<0.05; **P<0.01; ***P<0.001.

Table I. Primers for qPCR

Name	FOR	REV
**α-SMA**	GCTATTCAGGCTGTGCTGTC	GGTAGTCGGTGAGATCTCGG
**COL1A1**	CATGCCGTGACCTCAAGATG	TCCATCGGTCATGCTCTCTC
**β-actin**	TGTGTTGTCCCTGTATGCCT	AATGTCACGCACGATTTCCC
